# Influence of Unmodified and β-Glycerophosphate Cross-Linked Chitosan on Anti-Candida Activity of Clotrimazole in Semi-Solid Delivery Systems

**DOI:** 10.3390/ijms151017765

**Published:** 2014-09-30

**Authors:** Emilia Szymańska, Katarzyna Winnicka, Piotr Wieczorek, Paweł Tomasz Sacha, Elżbieta Anna Tryniszewska

**Affiliations:** 1 Department of Pharmaceutical Technology, Faculty of Pharmacy, Medical University of Białystok, Mickiewicza 2c, 15-222 Białystok, Poland; E-Mail: esz@umb.edu.pl; 2 Department of Microbiological Diagnostics and Infectious Immunology, Faculty of Pharmacy, Medical University of Białystok, Kilińskiego 1, 15-089 Białystok, Poland; E-Mails: piowie@umb.edu.pl (P.W.); sachpt@umb.edu.pl (P.T.S.); zdmik@umb.edu.pl (E.A.T.)

**Keywords:** chitosan, ion cross-linking, β-glycerophosphate, hydrogel, clotrimazole, anti-*Candida* activity

## Abstract

The combination of an antifungal agent and drug carrier with adjunctive antimicrobial properties represents novel strategy of complex therapy in pharmaceutical technology. The goal of this study was to investigate the unmodified and ion cross-linked chitosan’s influence on anti-*Candida* activity of clotrimazole used as a model drug in hydrogels. It was particularly crucial to explore whether the chitosans’ structure modification by β-glycerophosphate altered its antifungal properties. Antifungal studies (performed by plate diffusion method according to CLSI reference protocol) revealed that hydrogels obtained with chitosan/β-glycerophosphate displayed lower anti-*Candida* effect, probably as a result of weakened polycationic properties of chitosan in the presence of ion cross-linker. Designed chitosan hydrogels with clotrimazole were found to be more efficient against tested *Candida* strains and showed more favorable drug release profile compared to commercially available product. These observations indicate that novel chitosan formulations may be considered as promising semi-solid delivery system of clotrimazole.

## 1. Introduction

Chitosan is one of the natural multifunctional polymers, which due to its biocompatibility, mucoadhesiveness and penetration enhancement properties is expected to play promising role in biomedical and pharmaceutical field [1,2]. Chitosan—consisting of glucosamine and *N*-acetylglucosamine units—is obtained by deacetylation of chitin derived mainly from exoskeleton of crustaceans [3]. Recently, plenty of data has drawn attention to the use of chitosan semi-solid dosage forms in prolonged drug delivery [4,5,6]. Hydration of chitosan in acidic pH leads to form swellable matrix which hinder the entrance of water and as a result, prolongs the drug release rate. However, semi-solid drug carriers prepared with unmodified chitosan possess relatively low mechanical strength and acidic pH [4]. This acidic environment may lead to faster decomposition of active agents during storage of the dosage form. The physicochemical properties of chitosan semi-solid dosage forms may be improved by modifications of chitosan molecules using crosslinking agents [7,8]. Among various crosslinkers, β-glycerophosphate (β-GP) allows for the formation of homogeneous chitosan gel in mild conditions (without gel-like precipitate) with improved organoleptic properties [9]. It should also be pointed out that in combination with β-GP, chitosan becomes thermosensitive in diluted acids and can undergo gelation around body temperature [10]. These properties make chitosan/β-GP material a promising tool for a variety of applications, such as local drug delivery systems or injectable carriers for tissue-engineering [11].

In recent years, prevalence of mycotic infections has increased noticeably and antifungal resistance on conventional antifungal agents has been reported [12]. Hence, a particular effort in dosage forms technology should be made to evaluate novel therapeutic strategies in antifungal treatment. Polycationic polymers, like chitosan may provide the opportunity for combination therapy in which the polymer acts as the drug carrier and simultaneously as an active part of the therapy [13,14]. Nevertheless, it should be point out that the fungicidal activity of chitosan varies considerably according to a number of factors, namely hydrophilicity, molecular weight, modification of its structure, environmental pH, concentration of the polymer and the state of applied dosage form [15].

The data previously published by our group proved that chitosan as pharmaceutical excipient with favorable mucoadhesive properties and ability to form swellable control-release matrix can be successfully used for vaginal dosage forms with model antifungal agent [16,17]. However, potential interaction between conventional antifungal agents and chitosan—as antimicrobial adjunctive needs precise investigation, therefore the goal of this study was to explore the influence of chitosan in semi-solid delivery systems on pharmacological activity of clotrimazole against *Candida* species by using agar disc diffusion method. Clotrimazole—a broad-spectrum imidazole derivative effective against pathogenic dermatophytes and yeasts—was chosen as a model drug [18]. Considering that *Candida* species are common causative opportunistic pathogens and the prevalence of infections caused by this fungus has grown increasingly [19], *Candida albicans*, *Candida parapsilosis* and *Candida krusei* strains were selected for the experiments. Since to our knowledge, there are no studies devoted to anti-*Candida* activity of β-GP cross-linked chitosan, it was thus particularly crucial to investigate whether the modification of chitosan’s structure altered its antifungal activity. In the present work, the effect of chitosan/β-GP on the clotrimazole release profile from hydrogels was also examined.

## 2. Results and Discussion

### 2.1. Anti-Candida Activity of Chitosan Hydrogels

Sufficient treatment with imidazole derivatives, including clotrimazole necessitates multiple daily dosing and long-term therapy which is often inconvenient for patients. Moreover, *Candida* species as opportunistic pathogens are frequently clinically resistant to imidazole derivatives [20]. Chitosan—among other drug carriers—is regarded as useful compound in pharmaceutical technology due to its intrinsic antifungal and mucoadhesive properties. Chitosan itself at concentrations between 0.025% to 0.75% was found not to influence the viability of *Candida albicans* [14], whereas chitosan at higher concentration was demonstrated a significant antimicrobial activity [21]. In the present study, chitosan MMW with a degree of deacetylation 80% was employed in order to obtain different formulations of hydrogels with clotrimazole (hydrogels’ composition and preparation method are presented in the Experimental Section). During preliminary studies, two concentrations of chitosan (3% and 4%) assuring suitable viscosity of the hydrogels were chosen. Applying β-GP—a cross-linking agent with the mild alkalinity (p*K*_a_ = 6.34)—enabled to prevent precipitation or gelation of the polymer during hydrogel preparation.

**Figure 1:**
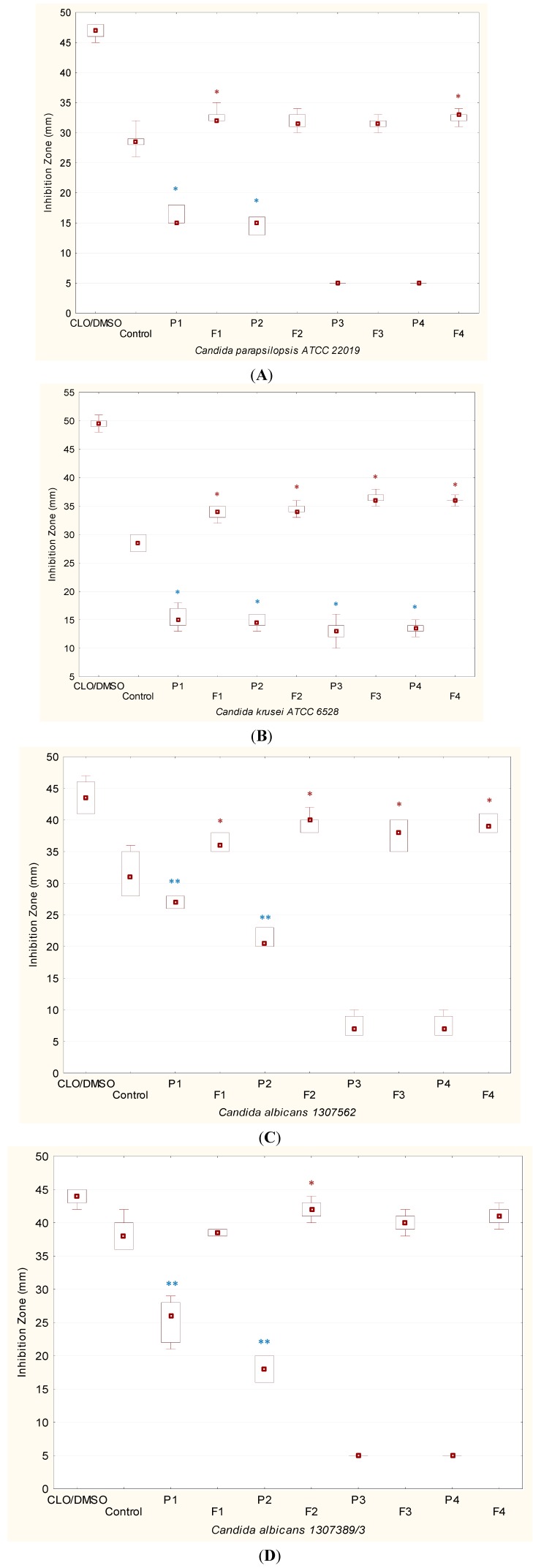
Box-plot graphs presenting antifungal activity of placebo hydrogels prepared with unmodified chitosan in concentration of 3% and 4% (P1, P2), with chitosan/β-GP in concentration of 3% and 4% (P3, P4), hydrogels with clotrimazole (CLO) prepared with unmodified chitosan (F1, F2), hydrogels with CLO prepared with chitosan/β-GP (F3, F4), reference standard (CLO/DMSO) and control (commercially available cream with CLO) against standard (**A**,**B**) or clinical strains of *Candida* sp. (**C**,**D**) (*n* = 3). Boxes contain 50% of all values and whiskers represent the 25th and 75th percentiles. Significantly different (*p* < 0.05) ratios are indicated by red asterisks (for hydrogels F1–F4) or by blue asterisks (for placebo hydrogels).

**Figure 2:**
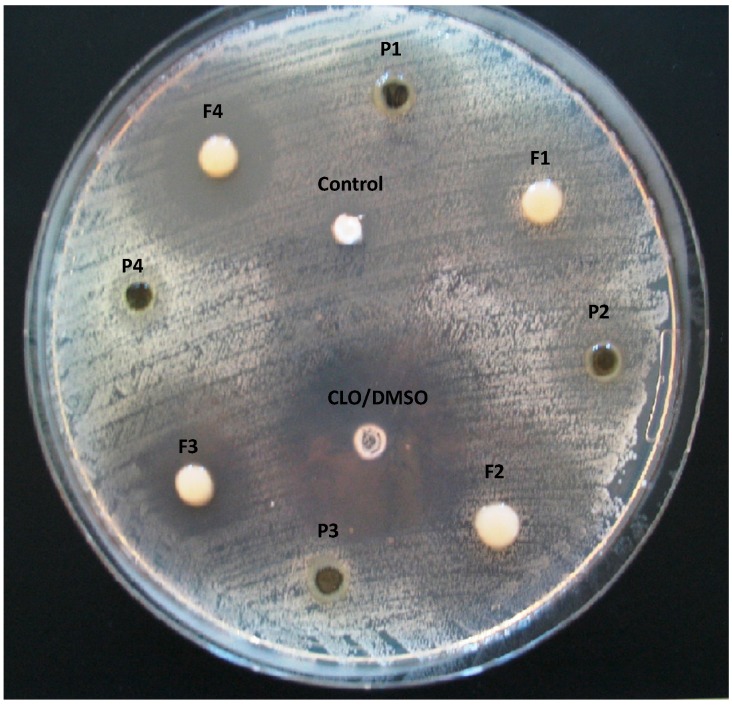
Representative image of plate diffusion test (after 24 h at 37 °C and 5% CO_2_) for *Candida albicans* 1307562 demonstrating the zone of inhibition around the wells containing: placebo hydrogels (P1–P4), unmodified chitosan and chitosan/β-GP hydrogels with clotrimazole (CLO) (F1–F4), control (commercially available cream with CLO) and reference standard (CLO/DMSO).

In the present study, the antifungal activity of chitosan modified by ion cross-linking on *Candida parapsilopsis*, *Candida krusei* and *Candida albicans* for the first time has been explored. In the performed experiments, 24 and 48 h were selected as the standard incubation time according to CLSI guidelines [22,23]. As no significant differences between chosen time points were noticed, the results of antifungal studies were presented after 24 h of incubation (Figure 1). Representative image of the plate diffusion test for *Candida albicans* 1307562 is shown in Figure 2.

It was found that placebo hydrogels with unmodified chitosan (P1 and P2) exerted a significant activity against all tested *Candida* strains (*p* < 0.05). Surprisingly, hydrogel P1 (with 3% of chitosan) was noticed to exhibit stronger effect in comparison to P2, probably because of its lower viscosity (Table 1) and better penetration through the culture medium.

**Table 1:** Viscosity at 25 °C of the performed hydrogels.

Formulation	Viscosity (mPa·s)
P1	17710.5 ± 85.7
P2	29302.5 ± 154.2
P3	19964.4 ± 114.6
P4	19297.6 ± 98.4
F1	19987.3 ± 108.2
F2	32048.1 ± 134.2
F3	20009.4 ± 109.4
F4	21828.1 ± 98.7

It was also shown that placebo hydrogels with β-GP cross-linked chitosan (P3 and P4) exerted markedly lower activity against tested *Candida* strains. The values of the inhibition zone after 24 h were found to be above 5 mm only for *Candida krusei* ATCC 6528 (13 ± 3 and 14 ± 1 mm for P3 and P4, respectively) (Figure 2B) and for *Candida albicans* 1307562 (7 ± 2 mm for P3 and P4) (Figure 1C). Similar effects of lowered antifungal activity were demonstrated when *N*-carboxyethylchitosan was studied as antimicrobial agent [24]. Reversely, Ji *et al.*, had shown that chitosan/β-GP hydrogel with chlorhexidine exhibited significant inhibitory activity against primary periodontal pathogens [25].

The obtained data might suggest that anti-*Candida* activity of chitosan is primarily dependent on its polycationic behavior. Diminished antifungal activity of placebo hydrogels P3 and P4 obtained by using β-GP might be a consequence of changing the p*K*_a_ of chitosan and declining in the number of protonated chitosan amino groups. Additionally, β-GP is known to create a protective hydration layer around the chitosan chains, which might be responsible for reduction of the interaction between chitosan and anionic components of *Candida* cell membrane [26]. Observed attenuation of antifungal activity may indicate the electrostatic interaction with yeasts anionic components as a fundamental chitosan mode of action. However, as the potency of anti-*Candida* activity of chitosan differed according to the evaluated *Candida* species, further studies are needed in order to verify β-GP cross-linked chitosan antifungal behavior.

In the present studies the influence of unmodified and β-GP cross-linked chitosan on the *in vitro* anti-*Candida* activity of clotrimazole in hydrogels was also evaluated. In comparison to commercially available product, a significant increase in activity of chitosan hydrogels with clotrimazole against tested *Candida parapsilosis* ATCC 22019, *Candida krusei* ATCC 6528 and *Candida albicans* 1307562 was observed. Certain differences in hydrogels antimicrobial activity according to the examined strains were noted. The highest zone inhibition (the range between 39 ± 1 to 42 ± 2 mm) was noticed in clinical strain of *Candida albicans* 1307389/3 (Figure 1D) and the lowest values—in *Candida parapsilopsis* ATCC 22019 (Figure 1A). Above results are in the agreement with previously reported data, in which anti-*Candida* activity of chitosan was found to be species-dependent [21]. The most effective in preventing growth of tested *Candida* strains was hydrogel F2.

### 2.2. In Vitro Release of Clotrimazole

**Figure 3:**
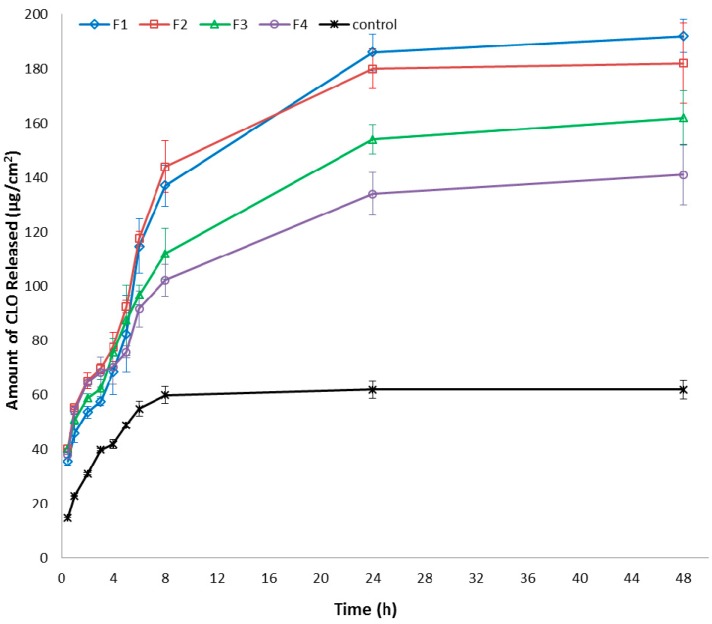
Amount of clotrimazole (CLO) per unit area (µg/cm^2^) released from different hydrogel formulations compared to commercially available product with clotrimazole (control).

As the drug release rate plays a significant role in the inhibition of fungi growth, the *in vitro* clotrimazole release from performed hydrogels was determined. As shown in Figure 3, the release profile was followed for 48 h and clotrimazole was definitely faster released from chitosan hydrogels than from commercially available product. After 6 h, the total amount of clotrimazole released from hydrogels with chitosan (F1 and F2) and from hydrogels with chitosan/β-GP (F3 and F4) was found to be 114.8 ± 11.9, 117.6 ± 9.4, 96.8 ± 9.4 and 91.7 ± 5.9 µg/cm^2^, respectively. For comparison, after 6 h of the study, the amount of clotrimazole released from commercially product was only 48.1 ± 2.1 µg/cm^2^. Higher drug release rate results in faster inhibition of the microbes growth and prevents the drug resistance [27]. Surprisingly, the amount of clotrimazole released was found not to be dependent on the concentration of chitosan used. It should be noted that formulations with β-GP cross-linked chitosan presented slightly lower clotrimazole release profile. The obtained results are in the agreement with the previously published data, in which modification of chitosan’s structure led to formation a thicker and less swellable matrix layer which hindered the entrance of water, and as a result, slower dissolution rate was observed [16].

## 3. Experimental Section

### 3.1. Materials

Chitosan (MMW—medium molecular weight with 80% of deacetylation degree—determined by titration method [28], viscosity of 1% solution in 1% acetic acid: 230 cP), β-glycerophosphate disodium salt hydrate, Cremophor EL, RPMI 1640 medium and dimethyl sulfoxide (DMSO) were purchased from Sigma-Aldrich (Steinheim, Germany). Clotrimazole was a gift sample from Ziaja Ltd. (Gdańsk, Poland). Glycerolum 86%, 80% acetic acid, sodium acetate, potassium dihydrogen phosphate, sodium hydroxide and disodium hydrogen phosphate were obtained from Chempur (Piekary Śląskie, Poland). d-Glucono-1,5-lactone and sodium benzoate—natural preservative was from Lonza (Basel, Switzerland). Stock cultures of *Candida parapsilopsis* ATCC 22019 and *Candida krusei* ATCC 6528 were provided by Microbiologics (St. Cloud, MN, USA). Cellulose acetate (CA) membrane filters (0.45 µm) were received from Millipore (Billerica, MA, USA) and Cuprophan^®^ from Medicell (London, UK). Commercially available cream with clotrimazole (2%) Glaxo SmithKline Pharmaceuticals (excipients: cetylstearyl alcohol, oktyldodecanol, polysorbate 60, sorbitan stearate, synthetic cetaceum, benzyl alcohol and purified water) was used as a control. Methanol was HPLC grade and it was purchased from Merck (Darmstadt, Germany). Water for HPLC was distilled and passed through a reverse osmosis system Milli-Q Reagent Water System (Billerica, MA, USA).

### 3.2. Preparation of Hydrogels

Hydrogels were prepared using mechanical stirrer model DT 200 (Witko, Łódź, Poland). Briefly, chitosan solutions were obtained by dissolving proper amount of chitosan in a gently heated acetic acid solution (1.8% and 2.4% *v*/*v*, respectively) and stirred until homogenous mixture appeared. The weight ratio chitosan:acetic acid (0.6:1.0) was selected to achieve total dissolution of chitosan [29]. Hydrogel pH was around 4.0. Additionally, to prepare cross-linked chitosan hydrogels, 45% (*w*/*w*) aqueous solution of β-GP was prepared and chilled along with the chitosan solution in the ice bath for 30 min. The appropriate amount of cold β-GP solution—necessary to adjust the pH of hydrogels to 4.5—was added dropwise to the cold chitosan solution with continuously stirring. Thus, 100 g of hydrogel contained 1.89 g (P3; F3) or 2.52 g (P4; F4) of β-GP with a constant weight ratio chitosan to β-GP 1:0.63. The final polymer concentrations were 3% or 4% (*w*/*v*). The composition of placebo hydrogels (P1–P4) and formulations with clotrimazole (F1–F4) is given in Table 2.

### 3.3. Test Organisms

The antifungal activity was performed against yeast cultures *Candida parapsilopsis* ATCC 22019, *Candida krusei* ATCC 6528, and against clinical strains belonging to the species *Candida albicans*. *Candida* strains were isolated from selected patients with candidiasis, identified morphologically [30], and frozen in potato dextrose broth at −70 °C. Preceding the antifungal susceptibility testing, each strain was inoculated on potato dextrose agar plates to ensure optimal growth characteristics and purity. Next, yeast cells were suspended in saline and adjusted spectrophotometrically to RPMI 1640 medium.

**Table 2:** Composition of prepared hydrogels.

Component (g)	Formulation							
	P1	P2	P3	P4	F1	F2	F3	F4
Clotrimazole	-	-	-	-	2.0	2.0	2.0	2.0
Chitosan	3.0	4.0	3.0	4.0	3.0	4.0	3.0	4.0
Glycerolum 86%	5.0	5.0	5.0	5.0	5.0	5.0	5.0	5.0
Cremophor EL	6.0	6.0	6.0	6.0	6.0	6.0	6.0	6.0
β-GP 45% (*w*/*w*)	-	-	4.2	5.6	-	-	4.2	5.6
d-Glucono-1,5-lactone and sodium benzoate	1.0	1.0	1.0	1.0	1.0	1.0	1.0	1.0
1.8% Acetic acid ad	100.0	-	100.0	-	100.0	-	100.0	-
2.4% Acetic acid ad	-	100.0	-	100.0	-	100.0	-	100.0

### 3.4. Plate Diffusion Method

For the investigation of the antimicrobial activity of hydrogels, the plate diffusion method was employed according to the Clinical and Laboratory Standards Institute (CLSI) guidelines [22,23]. The medium RPMI 1640 (with l-glutamine, without sodium bicarbonate, buffered to pH 7.0 with 3-(*n*-morpholino)propanesulfonic acid) was used for susceptibility testing. The initial density of *Candida* was approximately 2–5 × 10^6^ colony forming units (CFU)/mL. Inoculums of fungi (with density of 0.5 in McFarland scale) were prepared in sterile 0.9% NaCl solution and suspended in RPMI 1640 medium in a final density of 5 × 10^4^ CFU/mL. Then Petri dishes containing Sabouraud’s dextrose agar were seeded with 100 µL of the *Candida* inoculum. After the plates solidified at ambient temperature, a 5 mm diameter wells were cut in the inoculated agar plates and 100 mg of various formulations of chitosan hydrogels (which corresponded to 2 mg of active drug) were placed in each well. Solution of clotrimazole in DMSO (reference standard) and commercially available cream with clotrimazole were used as control. The growth of the tested strains was not influenced by the presence of acetic acid (data not shown). The plates were incubated at 37 ± 0.1 °C for 24 and 48 h. Antifungal activity was expressed as the mean of inhibition zones (mm)—measured with a caliper (Beta 161DGT, Sovico, Italy)—around each well with the accuracy of 0.1 mm [31,32]. All determinations were made in triplicate for each test microorganism.

### 3.5. Hydrogels’ Viscosity Measurements

The viscosity of the hydrogels was measured using a digital rotational Brookfield DV-III ULTRA Viscometer (Stuttgart, Germany). All measurements of apparent viscosity were carried out in a temperature-controlled environment at 25 °C. Hydrogels (0.5 g) were placed in the sampler holder of the viscometer and the C-52 spindle (with the shear rate of 10 s^−1^) was lowered into the sample. Each experiment was carried out three times.

### 3.6. HPLC Analysis

The concentration of clotrimazole in the medium was determined by the HPLC system Agilent Technologies 1200 equipped with a G1312A binary pump, a G1316A thermostat, a G1379B degasser and a G1315B diode array detector (Agilent, Waldbronn, Germany). Data collection and analysis were performed using Chemstation 6.0 software (Chemstation, Berlin, Germany). Isocratic separation was achieved on a Zorbax Eclipse XDB–C18, 4.6 × 150 mm, 5 μm column (Agilent, Waldbronn, Germany). Mobile phase was methanol-phosphate buffer pH 7.4 (4:1, *v*/*v*), the flow rate was 1.0 mL/min and UV detection was performed at a wavelength of 210 nm [33]. The column temperature was maintained at 25 °C. For injection into the HPLC system, 20 µL of sample was used. All reagents used for analysis were HPLC grade. The retention time of clotrimazole was 6.2 min. Standard calibration curve was linear over the range of 1–100 μg/mL (*R*^2^ = 0.995).

### 3.7. In Vitro Release of Clotrimazole

**Figure 4:**
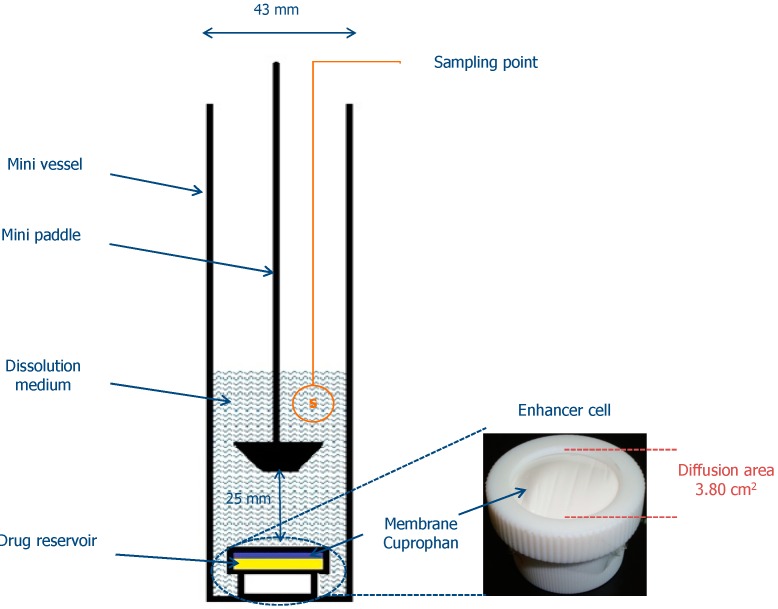
*In vitro* drug release study diagram.

*In vitro* release of clotrimazole was measured through natural cellulose membrane (Cuprophan, Medicell, London, UK) using an Enhancer cell (Agilent Technologies, Cary, NC, USA) with effective diffusion area of 3.80 cm^2^ [34,35]. The enhancer cell used in the study consisted of a teflon load ring, a cap, a membrane and a drug reservoir. About 2.0 g of each formulation was placed in the drug reservoir on the top of the membrane making certain that no entrapped air was present at the interface of semi-solid dosage form and the membrane. A USP dissolution Apparatus II (Agilent 708-DS, Agilent Technologies, Cary, NC, USA) equipped with mini vessels (250 mL) and mini paddles was used to measure the release of clotrimazole from the enhancer cell assembly [31]. A diagram illustrating the *in vitro* release study is presented in Figure 4. The dissolution medium was 100 mL of acetic buffer (pH 4.5) with addition of 1% surfactant maintained at 37 ± 0.5 °C and stirred at 75 rpm. Samples of 2 mL were withdrawn at the predetermined time intervals (0.5, 1, 2, 3, 4, 5, 6, 8, 24, 48 h), filtered through 0.45 µm CA paper filters, diluted with mobile phase and analyzed using the HPLC method (as described earlier). Withdrawn samples were replaced with equal volumes of the fresh medium. Sink conditions were maintained throughout the experiment. All release experiments were conducted in triplicate.

### 3.8. Statistical Analysis

Quantitative variables were expressed as the mean ± standard deviation and the median. A statistical analysis was performed using nonparametric technique the Kruskal-Wallis test with the Statistica 10.0 software (StatSoft, Kraków, Poland). Differences between groups were considered to be significant at *p* < 0.05.

## 4. Conclusions

Designed chitosan hydrogels are promising semi-solid delivery systems for clotrimazole as they showed enhanced anti-*Candida* activity and favorable drug release profile compared to commercially available product. Hydrogels obtained with cross-linked chitosan exhibited lower antifungal activity, which probably is a consequence of weakened polycationic properties of chitosan in the presence of β-GP. In order to provide more detailed data concerning the influence of β-GP crosslinking on chitosan antimicrobial activity, further study is needed.
